# Cleaning Plates after Soldering

**Published:** 1850-10

**Authors:** 


*Cleaning Plates after Soldering.*—Dr. S. F. Stearns, of Boston, recommends a method, and we believe it has also been adopted by others, by which the crystallized borax and discolorations of a plate after soldering, may be almost instantly removed. It consists in putting the piece, first, in a glass or porcelain vessel, with a sufficient quantity of water to cover it, then pouring in sulphuric acid until the discolorations on the plate disappear. The affinity of the acid for the water is such that the combination produces great heat, and it is this increase of the temperature of the mixture that causes it to act so promptly upon the crystallized borax and carbonaceous matter adhering to the plate. The same result will be produced by heating the mixture over a lamp, as is known to most jewellers and mechanical dentists.

